# Supplementary material for: Anticoagulant Treatment in Patients with AF and Very High Thromboembolic Risk in the Era before and after the Introduction of NOAC: Observation at a Polish Reference Centre
Source: Int J Environ Res Public Health. 2023 Jun 16;20(12):6145. doi: 10.3390/ijerph20126145 (PMC10298142; doi:10.3390/ijerph20126145)
Supplement: Supplementary file 1 [file ijerph-20-06145-s001.zip › ijerph-2360778-supplementary.pdf]

Supplementary Materials

Table S1a. Baseline characteristics of various CHA_2DS_2-VASc scale groups of females.

**CHA_2DS_2-VASc score**	**6 n=747**	**7 n=321**	**8 n=137**	**9 n=65**	**Total n=1270**
**Age (years) mean, (SD)**	79.6 (6.4)	80 (6.3)	81.7 (5.9)	83.8 (4.5)	80.1 (6.3)
**Age groups, n (%)**					
30-50	0 (0)	0 (0)	0 (0)	0 (0)	0 (0)
51-64	9 (1.2)	1 (0.3)	0 (0)	0 (0)	10 (0.8)
65-74	120 (16)	40 (12.5)	15 (10.9)	0 (0)	175 (13.8)
75-85	489 (65.5)	225 (70.1)	93 (67.9)	44 (67.7)	851 (67)
86-102	129 (17.3)	55 (17.1)	29 (21.2)	21 (32.3)	234 (18.4)
**Prior thromboembolism events**	134 (17.9)	172 (53.6)	137 (100)	65 (100)	508 (40)
**Hypertension**	700 (93.7)	296 (92.2)	130 (94.9)	65 (100)	1191 (93.8)
**Heart failure**	617 (82.6)	273 (85)	118 (86.1)	65 (100)	1073 (84.5)
**Diabetes mellitus**	348 (46.6)	189 (58.9)	75 (54.7)	65 (100)	677 (53.3)
**Vascular disease**	445 (59.6)	221 (68.8)	103 (75.2)	65 (100)	834 (65.7)

Table S1b. Baseline characteristics of various CHA_2DS_2-VASc scale groups of males.

**CHA_2DS_2-VASc score**	**5 n=723**	**6 n=305**	**7 n=115**	**8 n=28**	**9 n=0**	**Total n=1171**
**Age** (years) mean, (SD)	77 (8)	77 (7.7)	78.5 (6.3)	81.6 (4.6)	0 (0)	77.2 (7.7)
**Age groups, n (%)**						
30-50	2 (0.3)	0 (0)	0 (0)	0 (0)	0 (0)	2 (0.2)
51-64	37 (5.1)	22 (7.2)	0 (0)	0 (0)	0 (0)	59 (5)
65-74	203 (28.1)	64 (21)	22 (19.1)	0 (0)	0 (0)	289 (24.7)
75-85	377 (52.1)	186 (61)	82 (71.3)	20 (71.4)	0 (0)	665 (56.8)
86-102	104 (14.4)	33 (10.8)	11 (9.6)	8 (28.6)	0 (0)	156 (13.3)
**Prior thromboembolism events**	163 (22.5)	179 (58.7)	115 (100)	28 (100)	0 (0)	485 (41.4)
**Hypertension**	662 (91.6)	270 (88.5)	108 (93.9)	28 (100)	0 (0)	1068 (91.2)
**Heart failure**	624 (86.2)	270 (88.5)	103 (89.6)	28 (100)	0 (0)	1025(87.5)
**Diabetes mellitus**	333 (46.1)	195 (63.9)	55 (47.8)	28 (100)	0 (0)	611 (52.2)
**Vascular disease**	501 (69.3)	234 (76.7)	100 (87)	28 (100)	0 (0)	863 (73.7)

Table S2. The characteristics of non-OACs use in the entire population.

	**APT n=294**	**LMWH n=60**	**NONE n=82**
Sex (female), n (%)	156 (53.1)	28 (46.7)	43 (52.4)
Age, mean (SD)	79.6 (6.4)	76.9 (8.1)	81.5 (7.3)
Age < 65, n (%)	7 (2.4)	6 (10)	0 (0)
Age 65-74, n (%)	35 (11.9)	11 (18.3)	10 (12.2)
Age > 74, n (%)	252 (85.7)	43 (71.7)	72 (87.8)
**Clinical characteristics, n (%)**			
Heart failure, n (%)	263 (89.5)	57 (95)	73 (89)
Arterial hypertension, n (%)	270 (91.8)	53 (83.3)	75 (91.4)
Vascular disease, n (%)	196 (66.7)	38 (63.3)	46 (56.1)
Diabetes mellitus, n (%)	153 (52)	34 (59.7)	44 (53.7)
Previous stroke, n (%)	93 (31.6)	24 (40)	21 (25.6)
Previous TIA, n (%)	8 (2.7)	4 (6.7)	6 (7.3)
Other thromboembolic events, n (%)	6 (2)	4 (6.7)	2 (2.4)
Myocardial infarction, n (%)	98 (33.3)	14 (23.2)	23 (28)

PCI, n (%)	47 (16)	7 (11.7)	12 (14.6)
CABG, n (%)	14 (4.8)	2 (3.3)	6 (7.3)
PAD, n (%)	16 (5.4)	13 (21.7)	5 (6.1)
COPD, n (%)	39 (13.3)	8 (13.3)	9 (11)
Bleeding, n (%)	12 (4.1)	4 (6.7)	7 (8.5)
Peptic ulcer disease, n (%)	11 (3.7)	4 (6.7)	6 (7.3)
Cancer, n (%)	22 (7.5)	10 (16.7)	4 (5)
Thrombocythemia, n (%)	55 (18.7)	11 (18.3)	22 (26.8)
Anaemia, n (%)	68 (23.1)	24 (40)	34 (41.5)
Dialysis, n (%)	2 (0.7)	0 (0)	0 (0)
**Type of AF, n (%)**			
Paroxysmal, n (%)	151 (51.4)	25 (41.7)	39 (47.6)
Persistent, n (%)	5 (1.7)	2 (3.3)	2 (2.4)
Permanent, n (%)	138 (46.9)	33 (55)	41 (50)
**Thromboembolic risk**			
CHADS2, mean (SD)	3.9 (0.9)	4.2 (0.9)	3.9 (0.8)
CHA_2DS_2-VASc mean (SD)	6.1 (1)	6.2 (1)	6 (0.9)
CHA_2DS_2-VASc = 5, n (%)	84 (28.6)	14 (23.4)	29 (35.4)
CHA_2DS_2-VASc = 6, n (%)	133 (45.2)	29 (48.3)	31 (37.8)
CHA_2DS_2-VASc = 7, n (%)	54 (18.4)	10 (16.7)	15 (18.3)
CHA_2DS_2-VASc = 8, n (%)	13 (4.4)	5 (8.3)	7 (8.5)
CHA_2DS_2-VASc = 9, n (%)	10 (3.4)	2 (3.3)	0 (0)
**Bleeding risk**			
HAS-BLED, mean (SD)	2.3 (0.6)	2.6 (0.8)	2.5 (0.7)
HAS-BLED ≥ 3, n (%)	93 (31.6)	30 (50)	36 (43.9)
HAS-BLED ≥ 5, n (%)	0 (0)	1 (1.7)	0 (0)
**Laboratory test results**			
eGFR, mean (SD) (ml/min/1.73m^2)	49.1 (15.6) n=293	50.1 (23.5) n=59	46.2 (17.1) n=80
eGFR ≥ 60 ml/min/1.73m^2, n (%)	67 (22.9)	18 (30.5)	13 (16.2)
eGFR 59-45 ml/min/1.73m^2, n (%)	114 (38.9)	15 (25.4)	27 (33.7)
eGFR 44-30 ml/min/1.73 m^2, n (%)	79 (27)	14 (23.7)	29 (36.3)
eGFR 29-15 ml/min/1,73 m^2, n (%)	30 (10.2)	11 (18.7)	8 (10)
eGFR<15 ml/min/1,73 m^2, n (%)	3 (1)	1 (1.7)	3 (3.8)
**Echocardiography**			
EF mean (SD) (%)	45 (11.7) n=223	46 (10.2) n=48	45.2 (12) n=57
EF≥ 50%, n (%)	95 (42.6)	21 (43.8)	28 (49.1)
EF 41-49%, n (%)	41 (18.4)	17 (35.4)	5 (8.8)
EF≤40 %, n (%)	87 (39)	10 (20.8)	24 (42.1)
LA mean (SD) (mm)	44.6 (7) n=218	47.7 (8.3) n=47	46.8 (7.3) n=57
LA > 40 mm, n (%)	148 (67.9)	39 (83)	45 (79)
LA ≤ 40 mm, n (%)	70 (32.1)	8 (17)	12 (21)
**Reason for hospitalisation, n (%)**			
Electrical cardioversion, n (%)	2 (0.7)	1 (1.6)	1 (1.2)
Planned coronarography/PCI or ACS, n (%)	116 (39.5)	6 (10)	11 (13.4)
Planned CIED implantation/reimplantation, n (%)	89 (30.3)	15 (25)	19 (23.2)
Heart failure, n (%)	58 (19.7)	19 (31.7)	30 (36.6)
Ablation, n (%)	0 (0)	0 (0)	2 (2.4)
Other, n (%)	21 (7.1)	15 (25)	13 (15.9)
AF without any procedures, n (%)	8 (2.7)	4 (6.7)	6 (7.3)

Data are presented as number (percentage) or mean (standard deviation) (SD). Abbreviations: ACS, acute coronary syndromes; AF, atrial fibrillation; CABG; coronary artery bypass grafting; CAD, coronary artery disease; CIED, cardiac implantable electronic device; COPD, chronic obstructive pulmonary disease; eGFR, estimated Glomerular Filtration Rate; OAC, oral anticoagulants; PAD, peripheral artery disease; PCI, percutaneous coronary interventions; SD, standard deviation; TIA, transient ischaemic attack

Table S3. Predictors of lack of OAC prescription in high-risk patients

	OR	95% CI	p
Age (per year)	1.02	1.01-1.04	0.006
Age 65-74	0.58	0.43-0.78	**<0.001**
Age > 74	1.61	1.22-2.13	**0.001**
Hospitalisation	0.17	0.14-0.21	**<0.001**
**Clinical characteristics**			
Heart failure	1.61	1.15-2.26	**0.006**
Vascular disease	0.75	0.60-0.93	**0.008**
Stable CAD	0.74	0.59-0.93	**0.010**
PCI	0.59	0.45-0.79	**<0.001**
CABG	0.44	0.28-0.69	**<0.001**
PAD	0.51	0.35-0.73	**<0.001**
Cancer	2.03	1.36-3.05	**0.001**
**Type of AF**			
Paroxysmal	1.57	1.28-1.93	**<0.001**
Persistent	0.31	0.16-0.62	**0.001**
Permanent	0.76	0.62-0.94	**0.010**
**Bleeding risk**			
HAS-BLED (per unit)	0.80	0.69-0.93	**0.004**
HAS-BLED ≥ 3, n (%)	0.69	0.56-0.86	**0.001**
**Echocardiography**			
LA (mm)	0.97	0.95-0.98	**<0.001**
LA > 40 mm	0.48	0.36-0.64	**<0.001**
**Reason for hospitalisation**			
Electrical cardioversion	0.26	0.10-0.73	**0.010**
Planned coronary angiography/PCI or ACS	2.92	2.92-3.72	**<0.001**
Planned CIED implantation/reimplantation	0.72	0.57-0.92	**0.008**
Other	0.50	0.36-0.68	**<0.001**
AF without any procedures	0.60	0.36-0.99	**0.044**

Abbreviations: ACS, acute coronary syndromes; AF, atrial fibrillation; CABG; coronary artery bypass grafting; CAD, coronary artery disease; CIED, cardiac implantable electronic device; CI, confidence interval; OAC, oral anticoagulants; OR, odds ratio; PAD, peripheral artery disease; PCI, percutaneous coronary interventions
